# Dynamic modelling of high biomass density cultivation and biohydrogen production in different scales of flat plate photobioreactors

**DOI:** 10.1002/bit.25661

**Published:** 2015-07-14

**Authors:** Dongda Zhang, Pongsathorn Dechatiwongse, Ehecatl Antonio del Rio‐Chanona, Geoffrey C. Maitland, Klaus Hellgardt, Vassilios S. Vassiliadis

**Affiliations:** ^1^ Department of Chemical Engineering and BiotechnologyUniversity of CambridgePembroke StreetCambridgeCB2 3RAUK; ^2^ Department of Chemical EngineeringImperial College LondonSouth Kensington CampusLondonUK

**Keywords:** biohydrogen production, biomass cultivation, photo‐heterotrophic growth, dynamic simulation, light attenuation, photobioreactor

## Abstract

This paper investigates the scaling‐up of cyanobacterial biomass cultivation and biohydrogen production from laboratory to industrial scale. Two main aspects are investigated and presented, which to the best of our knowledge have never been addressed, namely the construction of an accurate dynamic model to simulate cyanobacterial photo‐heterotrophic growth and biohydrogen production and the prediction of the maximum biomass and hydrogen production in different scales of photobioreactors. To achieve the current goals, experimental data obtained from a laboratory experimental setup are fitted by a dynamic model. Based on the current model, two key original findings are made in this work. First, it is found that selecting low‐chlorophyll mutants is an efficient way to increase both biomass concentration and hydrogen production particularly in a large scale photobioreactor. Second, the current work proposes that the width of industrial scale photobioreactors should not exceed 0.20 m for biomass cultivation and 0.05 m for biohydrogen production, as severe light attenuation can be induced in the reactor beyond this threshold. Biotechnol. Bioeng. 2015;112: 2429–2438. © 2015 The Authors. *Biotechnology and Bioengineering* Published by Wiley Peiodicals, Inc.

## Introduction

Biohydrogen produced by green algae and cyanobacteria provide new opportunities to replace conventional fossil fuels and diversify sustainable energy sources (Mata et al., 2010). These microorganisms can convert solar energy to chemical energy for hydrogen production by different metabolic pathways such as photosynthesis and the nitrogen‐fixing process (Melis et al., 2000; Min and Sherman, 2010). Besides the application in renewable energy source production, microalgae and cyanobacteria are also cultivated as healthy food. For instance, microalgae such as *Spirulina* and *Chlorella* have been consumed as a food supplement in Mexico since antiquity, and are currently being cultivated for the commercialization of nutrient products in China, the United States and Thailand (Chu, 2012).

High cell density cultivation in photobioreactors (PBRs) greatly restricts the application of microalgae in both biofuels and biomass production (Brennan and Owende, 2010), and significant enhancements in the design of industrial photobioreactors have to take place in order to allow the large‐scale applicability of this process (Posten, 2009). To scale‐up biomass cultivation and biofuel production processes, the geometry of PBRs has to be well designed as it directly determines the length of the light path in the reactor and significantly affects biomass and biofuel productivity (Kumar et al., 2011; Posten, 2009; Tamburic et al., 2011; Ugwu et al., 2008). For example, in laboratory studies microalgal biomass concentration usually has an upper limit of 1 g L^−1^ – 3 g L^−1^ in photo‐autotrophic growth cultures. This is the result of light not penetrating the entire volume of PBRs beyond this biomass concentration and cells cannot receive sufficient light for their growth (Dechatiwongse et al., 2014; Tamburic et al., 2012; Wang et al., 2002).

In large‐scale research, biomass density is always much lower than that achieved at laboratory scale due to the larger reactor dimension which increases light attenuation in the reactors. For example, when the light path in an open pond increases from 0.10 to 0.30 m, the maximum biomass density of cyanobacterium *Anabaena* sp. is found to decrease from 0.55 g L^−1^ to 0.35 g L^−1^ (Clares et al., 2014; Moreno, 2003). The maximum biomass density of green alga *Chlorella ellipsoidea* is only found to be 0.53 g L^−1^ in a 200 L tubular PBR with a large diameter of 0.48 m (Wang et al., 2014). Even though CO_2_‐enriched air (1.5% CO_2_) was supplied to enhance biomass growth rate, recent work found that green algae such as *Nannochloropsis* sp. can only peak at a density of 2.48 g L^−1^ in a 1000 L flat plate PBR with a width of 0.10 m, and 1.20 g L^−1^ in a 500 L flat plate PBR with a width of 0.17 m (Cheng‐wu et al., 2001; Richmond and Cheng‐Wu, 2001). On the contrary, Tredici (Tredici et al., 1991) implemented a vertical panel PBR with a thickness of 0.016 m and found that both green algae and cyanobacteria can grow up to 4 – 7 g L^−1^ photoautotrophically, much higher than at laboratory scale. This indicates the importance of choosing and optimizing the dimensions of both flat plate and tubular PBRs for the industrialization of biomass cultivation.

Similarly, the width of PBRs also plays an essential role in biohydrogen production. In laboratory scale studies, biohydrogen productivity is determined to be 1.30 – 1.50 mL H_2_ L^−1^ (culture) h^−1^ from green alga *Chlamydomonas reinhardtii*, and 2.33 mL L^−1^ h^−1^ for cyanobacterium *Cyanothece* sp. in a 1 L flat plate PBR with a width of 0.025 m (Dechatiwongse et al., 2015; Tamburic et al., 2012, 2013). However, once the width increases up to 0.040 m, biohydrogen productivity from green algae *C. reinhardtii* and *C. noctigama* reduces to 0.97 mL L^−1^ h^−1^ and 0.56 mL L^−1^ h^−1^, respectively (Skjanes, 2008). In recent pilot‐scale studies where PBRs have a volume from 50 to 100 L, it is found that biohydrogen productivity with *C. reinhardtii* significantly reduces by 30.4% from 0.56 mL L^−1^ h^−1^ for PBR width 0.028 m to 0.17 mL L^−1^ h^−1^ when the width increases to 0.049 m (Giannelli and Torzillo, 2012; Scoma et al., 2012).

Despite its importance, little effort has been made in determining the optimal width of PBRs for biomass and biohydrogen production. The current study aims to propose strategies to improve the productivities of both biomass and biogas through optimization of the reactor width. Specifically, the nitrogen‐fixing cyanobacterium *Cyanothece* sp. ATCC 51142 is selected in this research, as this species is characterized by the highest hydrogen production rate of any other natural strain (Bandyopadhyay et al., 2010). As biohydrogen productivity is mainly achieved under cyanobacterial photo‐heterotrophic growth conditions where an additional carbon source such as glycerol is present, a dynamic model capable of simulating the entire *Cyanothece* sp growth and biohydrogen production process is constructed in the present study, which has to date not been considered in previous publications.

## Model Construction and Parameter Estimation

In this section, a novel dynamic model is constructed to simulate photo‐heterotrophic biomass growth and hydrogen production. Two aspects are significantly improved to guarantee the high accuracy of the current model. First, the proposed model takes into account the entire cyanobacterial growth phases observed in our experiments, from the growth phase where cells grow fast due to the presence of nutrients but hydrogen production is inhibited, to the decay phase where hydrogen is mainly generated but cells commence to die. Different limiting factors for cell growth and hydrogen production such as nitrate concentration and light intensity are included in the current model.

Second, a fourth order implicit method based on orthogonal collocation is selected in the parameter estimation process to guarantee high accuracy in the estimated parameter values in the current model (Hairer and Wanner, 1996). The use of the explicit Euler method which is mainly selected to discretize dynamic models in recent publications (Obeid et al., 2009; Wang et al., 2011; Xie et al., 2012) becomes unstable (Brugnano et al., 2009) with stiff highly‐nonlinear differential equations.

### Cyanobacterial Photo‐Autotrophic Growth Model

A previous study (Zhang et al., 2015) presents a dynamic model for the simulation of *Cyanothece* sp. photo‐autotrophic growth under nitrogen and carbon (CO_2_) sufficiency. Because the main focus of the current study is on the effects of light intensity and reactor configuration, temperature in the current experimental research is fixed at 35°C and its effects on cyanobacterial growth and decay rates are not considered. The detailed model is shown in Equations (1–3), and the parameters in the model are listed in Table I.

(1) $$\frac{dX}{dt}=\bar{k}(I)\cdot \mu_{\max,a}\cdot X(t)-\mu_{d,a}\cdot X^{2}(t)$$

(2) $$k(I)=\frac{I}{I+k_{s}+\frac{I^{2}}{k_{i}}}$$

(3) $$\bar{k}(I)=\frac{1}{L}\cdot \int_{0}^{L}k(I)dz=\frac{1}{L}\cdot \int_{0}^{L}\frac{I_{0}\cdot \exp\left[-\left(\tau_{c}\cdot X+\frac{3\cdot a_{g}}{d_{b}}\right)\right]}{I_{0}\cdot \exp\left[-\left(\tau_{c}\cdot X+\frac{3\cdot a_{g}}{d_{b}}\right)\right]+k_{s}+\frac{\left(I_{0}\cdot \exp\left[\left(\tau_{c}\cdot X+\frac{3\cdot a_{g}}{d_{b}}\right)\right]\right)}{k_{i}}}dz$$

where *τ_c_* is the light absorption coefficient by cyanobacteria. *μ_max,a_* and *μ_d,a_* are the maximum specific growth rate and decay rate, respectively. *I* is the local light intensity and *I*_0_ is the incident light intensity. *X* is biomass concentration. *k_s_* is a light saturation parameter for cell growth, and *k_i_* is a photoinhibition parameter for cell growth.

**Table I:** Parameters in the cyanobacterial photo‐autotrophic growth model

Parameter	Simulation result	Parameter	Simulation result
$\mu_{\max,\;a}$(hr^‐1^)	0.255	$d_{b}$ (m)	0.002
$\mu_{d,\;.a}$ (L hr^‐1^ g^‐1^)	0.00227	$\tau_{c}$ (m^2^ g^‐1^)	0.126
$\alpha_{g}$	0.0067	$k_{i}$ (μmol m^‐2^ s^‐1^)	457
$k_{s}$(μmol m^‐2^ s^‐1^)	165	L(m)	0.025

The above equations include both spatial variations (light transmission direction) and temporal variations as they constitute a dynamic model. To eliminate the spatial variation, the Trapezoidal rule is used to replace Equation (3) by Equation (4) in (Zhang et al., 2015). To ensure the accuracy of the results, the number of steps in the Trapezoidal rule is chosen as 20 resulting in a deviation of less than 10%, even when biomass concentration grows up to 12 g L^−1^ (Zhang et al., 2015).

(4) $$\bar{k}(I)=\frac{1}{L}\cdot \sum_{i=1}^{19}\left(k(I_{0})+2\cdot k(I_{i})+k(I_{20})\right)$$

### Cyanobacterial Photo‐Heterotrophic Growth and Hydrogen Production Model

In the present photo‐heterotrophic growth experiment, CO_2_ is replaced by glycerol as the latter compound was previously reported to provide electrons for hydrogen production and enhance the gas production rate (Bandyopadhyay et al., 2010). Glycerol is always present in excess and hence it is not a limiting factor. On the contrary, nitrate concentration is the limiting nutrient because it is essential for cyanobacterial growth but it can also inhibit the activity of nitrogenase which catalyses hydrogen production (Min and Sherman, 2010). Illumination in the PBR is another potential limiting factor for cell growth and gas production (Chen et al., 2011), as it is necessary to maintain biomass growth and hydrogen production.

The current work proposes a new dynamic model based on the Droop model, one of the most widely used models for bioprocess simulation (Vatcheva et al., 2006). The formulation of the present model is presented in Equations (5a–j). In the current experiment, cells continue growing for a while after the consumption of nitrate because of the accumulation of intracellular nitrogen when nitrate is present in the culture (Dechatiwongse et al., 2015). Therefore, the cell growth rate is a function of intracellular nitrogen concentration which is always represented by the nitrogen quota, the latter being defined as the ratio of intracellular nitrogen element mass to cell mass. Because in the current study nitrogen quota was not measured, a normalized nitrogen quota, defined as the ratio of nitrogen quota to maximum nitrogen quota when the culture is nitrate sufficient, has been used to replace the nitrogen quota in Equation (5a).

In this model, parameters in Equation (5g) have been estimated in previous work (Zhang et al., 2015), while other parameters need to be estimated in the current study. In Equation (7), the values of parameters in $\bar{k}(I)$ are the same as those in Table I as they are independent of cultivation mode (Feng et al., 2010), but the specific growth rate and decay rate have to be re‐estimated since they are remarkably enhanced after the supply of glycerol (Alagesan et al., 2013). The numerical integration method introduced in Section Cyanobacterial Photo‐Autotrophic Growth Model is also applied to approximate $\bar{h}(I)$, given in Equation (5h), and represents the effect of light intensity on hydrogen production.

(5a) $$\frac{dX}{dt}=\bar{k}(I)\cdot \mu_{\max,h}\cdot \left(1-\frac{k_{q}}{q}\right)\cdot X\cdot \frac{C}{C+K_{C}}-\mu_{d,h}\cdot X^{2}$$

(5b) $$\frac{dN}{dt}=-Y_{N/X}\cdot \bar{k}(I)\cdot \mu_{\max,h}\cdot \frac{N}{N+K_{N}}\cdot X$$

(5c) $$\frac{dq}{dt}=Y_{q/X}\cdot \bar{k}\left(I\right)\cdot \mu_{max,h}\cdot \frac{N}{N+K_{N}}-\bar{k}\left(I\right)\cdot \mu_{max,h}\cdot \left(1-\frac{k_{q}}{q}\right)\cdot q\cdot \frac{C}{C+K_{C}}$$

(5d) $$\frac{dH_{2}}{dt}=Y_{H/X}\cdot \bar{h}\left(I\right)\cdot X\cdot f(N)\cdot f(O)$$

(5e) $$\frac{dO_{2}}{dt}=Y_{O/X}\cdot \bar{k}\left(I\right)\cdot \mu_{max,h}\cdot \frac{N}{N+K_{N}}\cdot X-Y_{Od}\cdot \mu_{d,h}\cdot X^{2}\cdot \left(1-f(O)\right)$$

(5f) $$\frac{dC}{dt}=-Y_{C/X}\cdot \bar{k}\left(I\right)\cdot \mu_{max,h}\cdot \left(1-\frac{k_{q}}{q}\right)\cdot X\cdot \frac{C}{C+K_{C}}-Y_{C}\cdot X$$

(5g) $$h(I)=\frac{I}{I+k_{s,H2}+\frac{I^{2}}{k_{i,H2}}}$$

(5h) $$\bar{h}\left(I\right)=\frac{1}{L}\cdot \overset{19}{\underset{i=1}{\sum }}\left(h\left(I_{0}\right)+2\cdot h\left(I_{i}\right)+h\left(I_{20}\right)\right)$$

(5i) $$f\left(N\right)=0.5\cdot \frac{{\left({\left(N-100\right)}^{2}\right)}^{0.5}-(N-100)}{{\left({\left(N-100\right)}^{2}+0.1\right)}^{0.5}}$$

(5j) $$f\left(O_{2}\right)=1-\frac{O_{2}}{{\left({O_{2}}^{2}+0.1\right)}^{0.5}}$$

where $\mu_{max,h}$ and $\mu_{d,h}$ are the maximum specific photo‐heterotrophic growth and decay rate, respectively. q is the normalized nitrogen quota. $k_{q}$ denotes the normalized minimum nitrogen quota. N, C, $H_{2}$, and $O_{2}$ are the concentration of nitrate, glycerol, hydrogen, and oxygen, respectively. $K_{N}$ and $K_{C}$ are the half‐velocity constant of nitrate and glycerol, respectively. $Y_{q/X}$, $Y_{N/X}$, $Y_{C/X}$, $Y_{O/X}$. $Y_{H/X}$, $Y_{C}$, $Y_{Od}$ are the yields of nutrients and products as indicated by the respective subscripts of the symbols used. $k_{s,H2}$ and $k_{i,H2}$ are the light saturation and photoinhibition terms, respectively, for hydrogen production.

In Equation (5e), the oxygen generation rate is assumed to be a function of nitrate concentration instead of nitrogen quota. This is because the cyanobacterial nitrate uptake process requires a high amount of ATP which has to be synthesized through the photosynthesis pathway where molecular oxygen is generated (Min and Sherman, 2010). Once nitrate is consumed, the nitrate uptake metabolic pathway is stopped and oxygen concentration drops to zero. In Equation (5f), the first term on the right represents the consumption rate of glycerol for cell growth, and the second term on the right denotes the consumption rate of glycerol for cell maintenance. The consumption rate of glycerol for hydrogen production is not included in this equation since it is negligible compared to those for cell growth and maintenance. A cell decay term is also not included in Equation (5f) since it is assumed that glycerol consumption is independent of cell decay; otherwise an increase on glycerol concentration will be estimated based on Equation (5f) when cells start to decay, which is not physically correct. Two switch functions (Zhang et al., 2015), given in Equations (5i) and (5j), are applied to mediate the start and termination of hydrogen production as nitrogenase (the enzyme catalyzing the reduction of protons) is only activated when the culture is anaerobic and nitrate concentration is lower than 100 mg L^−1^ (Dechatiwongse et al., 2015; Min and Sherman, 2010).

To estimate the parameters for the model in Equations (5a–j), the orthogonal collocation method is selected to discretize the current model. The implementation for the optimization problems shown in this work is done in Pyomo, a Python‐based optimization environment. Specifically, Pyomo (Hart et al., 2012), a tool package for modelling optimization applications in Python, is used in this work to discretize and optimize dynamic parameter estimation problems. The specific nonlinear programming problem (NLP) solver used as a library in Pyomo to carry out the optimization is IPOPT (Wächter and Biegler, 2005).

**Figure 1:**
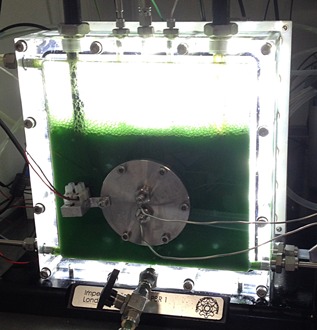
Flat‐plate photobioreactor used in experiments in this work.

In the current research, the laboratory scale photobioreactor is a 1 L flat‐plate photobioreactor with a width of 0.025 m (Tamburic et al., 2011). Illumination is provided through one surface (0.04 m^2^), as shown in Figure 1. The current experimental data are used to estimate the parameters in this model. The detailed of the current experimental work and reactor used can be found in (Dechatiwongse et al., 2015; Tamburic et al., 2011).

## Results and Discussion

### Results of Parameters

**Figure 2:**
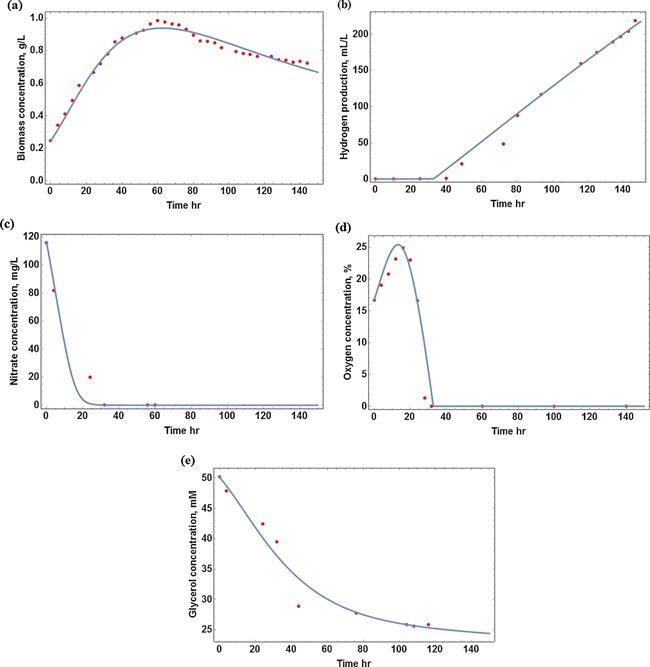
Comparison of simulation and experimental results for cyanobacterial photo‐heterotrophic growth and hydrogen production. **a:** biomass concentration; **b:** hydrogen yield; **c:** nitrate concentration; **d:** oxygen concentration; **e:** glycerol concentration.

Table II lists the parameters estimated in the current research. From Figure 2, it can be seen that the current model can represent the highly nonlinear features of the dynamic system well. It is not surprising that $K_{C}$ equals zero, meaning that cell growth and hydrogen production are not affected by glycerol concentration, as glycerol is always in excess and is not a limiting factor.

**Table II:** Parameters in the hydrogen production model

Parameter	Value	Parameter	Value
$\mu_{\max\text{,}\;a}$ (h^‐1^)	0.332	$K_{N}$ (mg)	50.0
$\mu_{d\text{,}\;.a}$ (L h^‐1^ g^‐1^)	0.00716	$Y_{N/X}$ (mg g^‐1^)	492.7
$k_{q}$	0.165	$Y_{q/x}$ (g^‐1^)	0.0317
$k_{s,\;H2}$ (μmol m^‐2^ s^‐1^)	140	$Y_{H/X}$ (mL g^‐1^ h^‐1^)	14.20
$k_{t,\;H2}$ (μmol m^‐2^ s^‐1^)	457	$Y_{O/X}$ (L g^‐1^)	81.02
$Y_{Od}$(L g^‐2^)	486.03	$Y_{c/x}$ (mmol g^‐1^)	20.454
$Y_{c}$ (mmol g^‐1^ h^‐1^)	0.0301	$K_{c}$ (mmol L^‐1^)	0.0

### Sensitivity Analysis

Sensitivity analysis is widely used in this work to explore the variability of model outputs (process substrates and products) with respect to changes in model inputs (parameters in dynamic models) (Fouchard et al., 2009). The normalized sensitivity as defined in Equation (6) (Morbidelli and Varma, 1988), signifies the normalized change of model outputs (y) with respect to the normalized change of the parameters (x). A positive sensitivity value indicates that an increase in x results in an increase in y, while a negative sensitivity value indicates that increasing x will lead to a decrease in y.

(6) $$S_{y/x}=\frac{dy}{dx}\cdot \frac{x}{y}$$

where y is the model output and x is any parameter in the overall process model.

**Figure 3:**
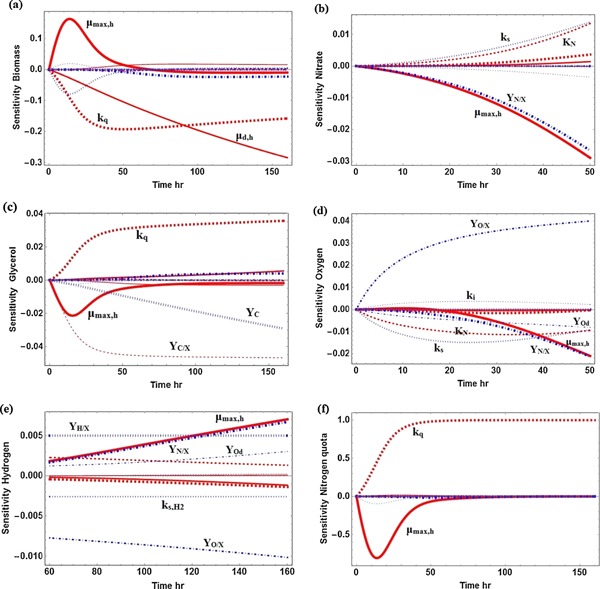
Sensitivity analysis of model output with respect to model input. **a:** biomass concentration; **b:** nitrate concentration; **c:** glycerol concentration; **d:** oxygen concentration; **e:** hydrogen production; **f:** nitrogen quota. The process duration is 150 h. As oxygen and nitrate are consumed within the first 50 h Figures 3(b) and (d) terminate at this time and Figure 3(e) starts at the 60th hour.

Figure 3 shows the sensitivity of substrates (nitrogen quota, nitrate, and glycerol) and products (biomass, hydrogen, and oxygen) with respect to model parameters. From Figure 3, it can be deduced that most of the model outputs (nitrate [Fig. 3b], glycerol [Fig. 3c], oxygen [Fig. 3d], and hydrogen [Fig. 3e]) are not sensitive to any model parameters. The normalized sensitivities of these outputs are less than ± 0.04, which means a 1% change of model input (parameter) can only lead to a very small change of less than 0.04% in the output.

On the contrary, biomass concentration (Fig. 3a) and nitrogen quota (Fig. 3f) have great sensitivities with respect to model parameters. A 1% change on model input can cause a change higher than 0.1% on these outputs. Both $\mu_{max,h}$ and $k_{q}$ are found to affect significantly biomass concentration and nitrogen quota. Based on Figure 3a, $\mu_{max,h}$ should be increased while $k_{q}$ should be decreased to enhance biomass production. This is logical as increasing $\mu_{max,h}$ increases the biomass growth rate and decreasing $k_{q}$ can extend the duration of the cyanobacterial growth phase.

Overall, from the current sensitivity analysis it can be concluded that if the process aim is to maximize biohydrogen production, more effort should be concentrated in seeking optimal operating conditions as gas productivity is not sensitive to cyanobacterial kinetic properties. On the other hand, if the main target of the process is biomass production, with hydrogen only a by‐product, it is essential to develop a mutant strain capable of growing rapidly and being more tolerant to a nitrogen‐deprived environment.

### Effects of Light Attenuation on Cell Growth and Hydrogen Production

**Figure 4:**
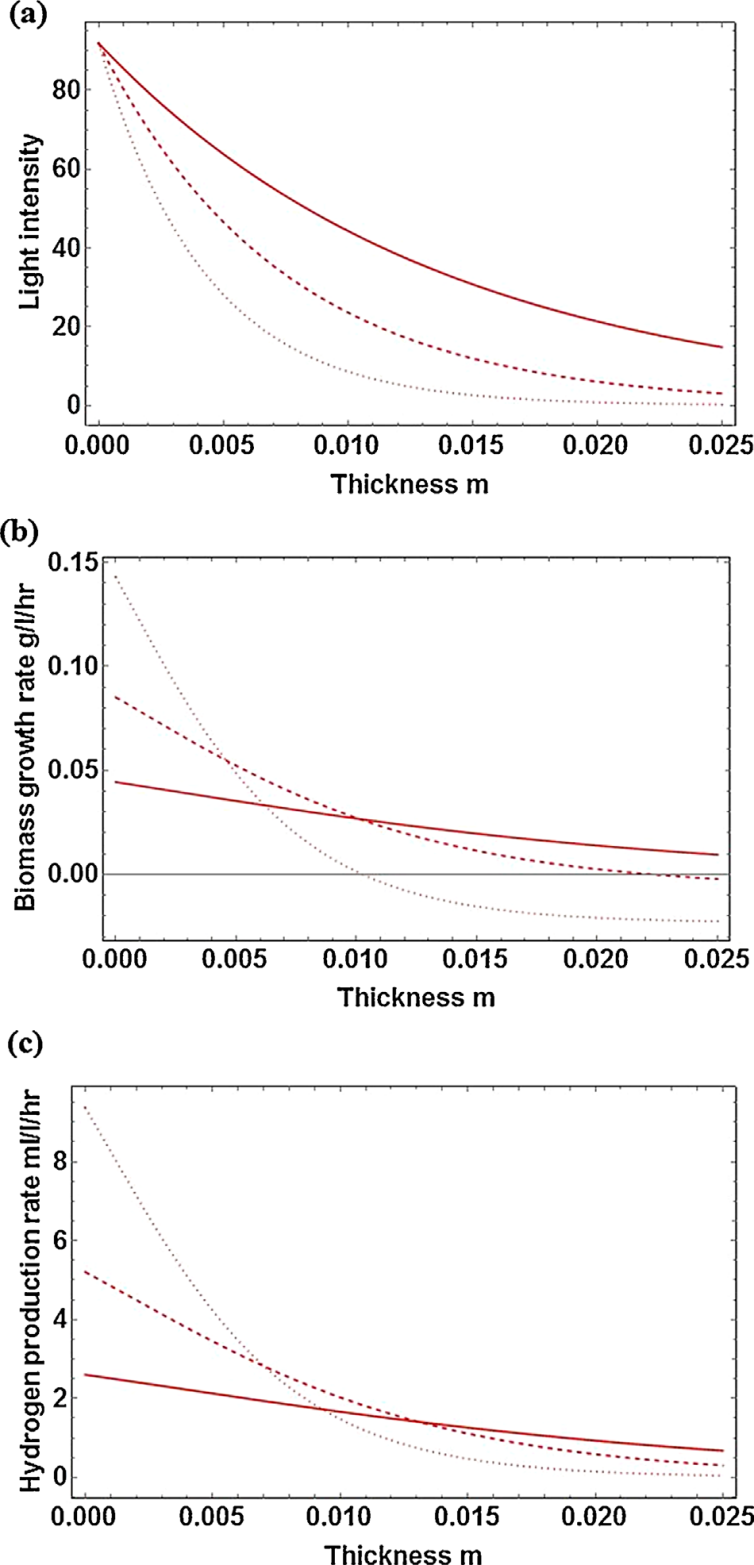
**a:** Local light intensity (µmol × m^−2 ^× s^−1^), **b:** cell growth rate and, **c:** hydrogen production rate in the current PBR with different biomass concentration. Solid line: biomass concentration of 0.5 g L^−1^, dashed line: biomass concentration of 1.0 g L^−1^, dotted line: biomass concentration of 1.8 g L^−1^. Incident light intensity is 92 µmol × m^−2 ^× s^−1^. The exposure surface is defined as the front surface (0.0 in *x*‐axis) and the other surface is defined as the back surface (0.025 in *x*‐axis).

Light attenuation in a PBR is mainly caused by bubble scattering and cell absorption (Zhang et al., 2015). Under the photo‐heterotrophic growth conditions studied, it is found that cell absorption is the primary factor causing light attenuation during the fermentation stage as the value of this term in Equation (3), $\tau_{c}\cdot X$, is 7 times higher than that of the bubble reflection term ($3\cdot \alpha_{g}/d_{b}$) even if biomass concentration is low (0.5 g L^−1^). Figure 4 shows the local light intensity, local cell growth rate, and local hydrogen production rate at different biomass concentrations in the present laboratory scale PBR.

When biomass concentration is low (0.5 g L^−1^), the distributions of the cyanobacterial growth rate and the hydrogen production rate in the reactor are relatively uniform (solid lines in Figs. 4b and c). At an intermediate biomass concentration (1.0 g L^−1^), both the cell growth rate and the hydrogen production rate are higher than those at low biomass concentration in the major part of the reactor, since more cells are exposed to illumination. However, because of the increased light attenuation (Fig. 4a) local light intensity in the back part of the reactor is significantly reduced, which leads to a slightly lower cell growth rate and hydrogen production rate compared to those at the low biomass concentration in the back zone (dashed lines in Figs. 4b and 4c).

If biomass concentration is high (1.8 g L^−1^), both rates are significantly improved in the front part of the reactor. This is due to the fact that local illumination intensity in the front part of the reactor is not much affected by light attenuation as it is proximity to the light source. As biomass growth and hydrogen production rates depend on the biomass concentration (Equations [5a] and [5d], respectively), both rates increase with the increasing biomass concentration. The major volume of the reactor, however, is subject to the severe photo‐limiting environment, and both rates are much lower than those at the low biomass concentration (dotted lines in Figs. 4b and c). Local hydrogen production rate drops to zero at the back of the reactor where cell growth rate even becomes negative, indicating that the local illumination is too low to maintain cell growth and hydrogen production. In this case, the photobioreactor is not well‐utilized as only part of it is functional.

In fact, as hydrogen is mainly generated in the cyanobacterial stationary and decay phases (Dechatiwongse et al., 2015), biomass concentration usually keeps decreasing when the gas is produced. In order to guarantee that the entire volume of the photobioreactor is viable for gas production, the maximum biomass concentration should be controlled around 1.0 g L^−1^ in the current PBR. Beyond this value, light is not able to penetrate the entire volume of the reactor. However, due to the fact that light does not directly participate in the enzymatic reaction of hydrogen production, cyanobacteria can still generate hydrogen in dark if they have experienced sufficient illumination to store ATP for cell growth and hydrogen production. As a result, the current simulation work may underestimate the maximum biomass concentration in case the culture mixing rate along the light transmission direction in the reactor is higher than the enzymatic reaction rate (Huang et al., 2014).

### Biomass Cultivation and Hydrogen Production of Low‐Chlorophyll Content Mutants

From the sensitivity analysis, it is found that identifying the optimal operating conditions is essential for hydrogen production. Because of the cost for an additional carbon source (e.g., glycerol), the photo‐autotrophic growth culture is mainly selected for biomass cultivation. Therefore, in the current study, for biomass cultivation the photo‐autotrophic growth model will be selected and for hydrogen production the photo‐heterotrophic growth model will be chosen.

Low‐chlorophyll mutants of *Dunaliella tertiolecta* have been isolated recently (Bibby, 2014). These mutants are able to operate with a lower functional absorption cross‐section photosystems, and are capable of reducing light attenuation as they absorb less light energy compared to the wild‐type strain. Although a mutant of *Cyanothece* sp. ATCC 51142 with similar features has not been created yet, it is useful to predict the performance of such a mutant in terms of biomass cultivation and hydrogen production. Since the mutants of *Dunaliella tertiolecta* were found to contain reduced cross‐section of photosystems of 30–40%, the current study assumes that a hypothetical low‐chlorophyll *Cyanothece* sp. mutant may also be created with a reduced cross‐section of 30%.

Previous research has demonstrated that parameters in the Aiba model have their specific biological meaning (Zhang et al., 2015). The Aiba model in Equation (2) can be rewritten as in Equation (7). From this, it is concluded that the change of cross‐section of the photosystem unit (PSU) will affect the values of $k_{s}$, $k_{is}$, $k_{s,H2}$, and $k_{i,H2}$. Furthermore, as the cyanobacterial light extinction coefficient ($\tau_{c}$) is proportional to the mass fraction of chlorophyll in cells, its value will also be reduced in a low‐chlorophyll mutant.

(7) $$k\left(I\right)=\frac{I}{I+k_{s}+\frac{I^{2}}{k_{i}}}=\frac{I\cdot \frac{1}{v\cdot \sigma }}{I+\frac{1}{K\cdot \sigma }+\frac{I^{2}}{\frac{1}{v\cdot \sigma }}}$$

where σ is the cross‐section of PSU unit, m^2^ J^−1^; v is the equivalent turnover time of PSU, s^−1^; K is the ratio of the protein damage constant to the protein recovery constant, s^−1^.

By re‐calculating the values of parameters in the Aiba model, the present simulation results demonstrate that the mutant could increase biomass concentration by 14% under photo‐autotrophic growth condition. Because of the reduction of light attenuation, illumination distribution is much improved in these reactors. Furthermore, the mutant would offer a significantly enhanced hydrogen productivity of 24% when the width of the reactor is scaled up to 0.2 m. As light attenuation is the main factor limiting hydrogen production in a large scale reactor, such a mutant would show potential for industrial biohydrogen production.

Despite the potential for large‐scale PBR implementation, the gains observed in a laboratory environment would not be large. In fact, if the reactor width is further reduced in the current laboratory scale PBR, this mutant would exhibit lower hydrogen productivity as the primary limiting factor for hydrogen production is no longer light attenuation. The better light distribution in the laboratory scale reactor (e.g., 0.01 m) can enhance cell growth rate, which accelerates the consumption of nitrogen quota. As a result, cell decay due to the lack of nitrogen source will be enhanced and the hydrogen production rate will be reduced. Therefore, a laboratory scale PBR might not show the potential enhancements possible at industrial scale, that is, the potential benefits might be simply overlooked as there is usually no scale‐up assessment carried out when collecting laboratory data. This fact is also consistent with the conclusion from sensitivity analysis that identifying the optimal operating conditions is more important for a small‐scale process.

### High Cell Density Cultivation and Hydrogen Production in Large‐Scale PBRs

To explore further the effects of PBR configuration on biomass cultivation and hydrogen production, the current work calculates the maximum biomass concentration and average hydrogen production rate in different configurations of PBRs and compares them in Table III.

**Table III:** Comparison of cyanobacterial maximum biomass concentration and average hydrogen production rate in different scales of flat‐plate PBRs

I_0_‐n‐d	C_max_ (g L^−1^)	I_0_‐n‐d	C_max_ (g L^−1^)
457‐1‐0.025	6.22	114‐2‐0.20	2.67
457‐1‐0.10	4.23	229‐2‐0.20	2.85
457‐1‐0.20	2.74	457‐2‐0.20	2.96
457‐1‐0.50	1.58	914‐2‐0.20	3.21
I_0_‐n‐d	H_2_ (mL L^‐1^ hr^‐1^)	I_0_‐n‐d	H_2_ (mL L^‐1^ hr^‐1^)
457‐1‐0.025	4.61	114‐2‐0.20	0.65
457‐1‐0.05	2.24	229‐2‐0.20	0.96
457‐1‐0.1	0.97	457‐2‐0.20	1.26
457‐1‐0.2	0.37	914‐2‐0.20	1.53

The maximum biomass concentration is based on the assumption of photo‐autotrophic growth. I_0_ represents incident light intensity (µmol m^−2^ s^−1^) on each exposure surface, n represents the number of exposure surfaces, d is the reactor thickness (m). The average hydrogen production rate is calculated with initial biomass concentration set as 3.5 g L^−1^ and batch process time of 240 h. C_max_ is the predicted maximum biomass concentration (g L^−1^), and H_2_ represents the average productivity of hydrogen (mL L^−1^ h^−1^).

For biomass cultivation, it is found that the reactor width should not exceed 0.2 m, as the maximum biomass concentration is severely reduced (half compared to a width of 0.025 m). With regards to incident light intensity, using a low intensity on two exposure surfaces is a better strategy to enhance biomass concentration and save lighting costs. For hydrogen production, very similar observations are made. However in this case the width should not be greater than 0.05 m (half compared to a width of 0.025 m) as the metabolic pathway of hydrogen production is more energy demanding and a uniform light distribution is vital.

## Conclusion

This contribution presents the detailed modelling of cyanobacterial photo‐heterotrophic growth and hydrogen production. To the best of our knowledge, it is the first time a dynamic model is presented, which is capable of simulating all phases of this bioprocess, and also includes the effects of limiting nutrient concentration, light intensity, and light attenuation.

Based on a sensitivity analysis, it is shown that identifying the optimal operating conditions is more effective than developing mutants for increasing hydrogen production in a laboratory scale reactor.

Cell absorption has been determined to be the primary limiting factor for light attenuation in the present laboratory scale PBR. It is found that cultivating a low‐chlorophyll mutant is an efficient way to enhance both biomass and hydrogen productions in a large‐scale PBR, although this effect might not be observed at laboratory scale. Finally, for the design of pilot‐scale PBRs, the present research calculates the maximum biomass concentration and the average hydrogen productivity in different configurations of PBRs. It is concluded that using lower incident light intensity but double exposure surfaces is a better strategy for the simultaneous production of biomass and biohydrogen.

D. Zhang gratefully acknowledges the support from his family. P. Dechatiwongse is supported by a scholarship from the Royal Thai Government, Thailand. The Solar Hydrogen Project was funded by the UK Engineering and Physical Sciences Research Council (EPSRC), project reference EP/F00270X/1. E. A. del Rio‐Chanona is funded by CONACyT scholarship No. 522530 from the Secretariat of Public Education and the Mexican government.
